# Title TBD

**DOI:** 10.1002/alz70858_106601

**Published:** 2025-12-25

**Authors:** Matthew A Jamieson, Meiyii Lim, Bruce W. Wilson, Lynsey A Stewart, Dhai Al Yayyai, Ruth Aylett, Matthew Aylett, Mario A A Parra

**Affiliations:** ^1^ University of Strathclyde, Glasgow, United Kingdom; ^2^ Heriot‐Watt University, Edinburgh, United Kingdom; ^3^ Heriot‐Watt University, EDINBURGH, United Kingdom; ^4^ University of Strathcylde, Glasgow, Glasgow, United Kingdom

## Abstract

**Background:**

Reminiscing can improve well‐being in people with dementia, and technology offers new ways to support this (Macleod et al., 2021). AMPER (Agent‐based Memory Prosthesis to Encourage Reminiscing) is a digital tool that uses personalised image, audio, and video cues, combined with prompts from a digital character, to facilitate reminiscence (M. Aylett et al., 2024a&b; Wilson et al., 2024). AMPER aims to provide cues and prompts for preferred topics within the user's reminiscence bump (ages 10–30).

**Methods:**

Eighteen pairs of healthy older adults experienced using AMPER for the first time, aiming at a) evaluating use and acceptance to inform app development prior to a randomised controlled trial and b) examining how cognitive ability and self‐reported autobiographical memory (AM) ability impacted technology acceptance and interaction quality, as measured by the length time taken during use. One participant in each pair pre‐specified their interests, and the pair reminisced about three ‘stories.’ Each story included three images from the BBC Reminiscence Archive (‘cues’) and AI‐generated questions (‘prompts’) spoken by the digital character. A semi‐structured interview followed, along with questionnaires on technology acceptance (ALMERE), self‐reported AM ability (SAM and ART), and cognitive ability (ACE‐III).

**Results:**

Participants spent an average of 4 minutes 3 seconds (SD = 3min34s) reminiscing per story. People found the technology easy to use (ALMERE ease of use items median rating was 4 out of 5 (range=3). Interviews identified key app and procedure improvements: giving the character more of a personality, making prompts less specific to the images, and ensuring user expectations align with app functionality. Significant correlations were found between neither technology acceptance and time spent with each story nor acceptability and self‐rated memory or cognitive ability.

**Conclusions:**

AMPER is usable and acceptable among healthy older adults. This preliminary study has refined the AMPER system and procedures for an upcoming randomised controlled trial. This will investigate whether these user acceptance findings replicate in people with dementia and whether AMPER can improve AM ability over time.

**References**

1. Aylett et al. (2024a). https://doi.org/10.1145/3652988.3673964

2. Aylett et al. (2024b). https://ojs.aaai.org/index.php/AAAI‐SS/article/view/31781

3. Macleod et al. (2021). https://doi.org/10.1177/1471301220941275

4. Wilson et al. (2024). https://doi.org/10.1145/3652988.3696201

